# Application of Complementary and Alternative Medicine on Neurodegenerative Disorders 2013

**DOI:** 10.1155/2014/463929

**Published:** 2014-01-22

**Authors:** Paul Siu-Po Ip, Karl Wah-Keung Tsim, Kelvin Chan, Rudolf Bauer

**Affiliations:** ^1^School of Chinese Medicine, The Chinese University of Hong Kong, Shatin, New Territories, Hong Kong; ^2^Division of Life Science, The Hong Kong University of Science and Technology, Clear Water Bay, Kowloon, Hong Kong; ^3^Faculty of Pharmacy, The University of Sydney, NSW 2006, Australia; ^4^Centre for Complementary Medicine Research, University of Western Sydney, NSW 2560, Australia; ^5^Department of Pharmacognosy, Institute of Pharmaceutical Sciences, University of Graz, 8010 Graz, Austria

Neurodegenerative disorders such as Alzheimer's disease and Parkinson's disease are characterized by progressive loss of neurons in sensory, motor, and cognitive systems. Based on limited knowledge on the pathogenic mechanisms of the diseases, some drugs have been developed. For example, acetylcholinesterase inhibitors and N-methyl-D-aspartate receptor antagonist have been widely used for treating Alzheimer's disease. However, these drugs have not shown promising results and tolerance may be developed after a short period of treatment. The etiopathology of neurodegenerative diseases is extremely complex and has not been fully revealed. It is reasonable to expect that drugs acting on multiple targets can provide better treatment results than those acting on a single target for these diseases. Some herbal remedies have been used traditionally for improving cognitive function and treating mental diseases for many years. These drugs contain a number of active ingredients and can be the potential candidates for the treatment of neurodegenerative disorders.

In the special issue of “Application of Complementary and Alternative Medicine on Neurodegenerative Disorders” published last year, we have collected papers on the application of complementary and alternative medicine for treating Alzheimer's disease, Parkinson's disease, and depression. In this year, Alzheimer's disease is still a hot topic of investigation. Alzheimer's disease is a well-known neurodegenerative disease characterized by a progressive deterioration of cognitive function and memory. Although the pathological mechanism of the disease is not fully understood, the deposition of beta-amyloid and the generation of reactive oxygen species are believed to play important roles in the pathogenesis of the disease. Therefore, neurotoxicity induced by beta-amyloid or oxidants has been commonly used as a cellular model of Alzheimer's disease. In this issue, C.-F. Ng et al. reported that a decoction of Gastrodiae Rhizoma (rhizome of *Gastrodia elata* Bl.) was able to protect against *in vivo* and *in vitro* neurotoxicity induced by beta-amyloid through the inhibition of apoptosis and oxidative damage. A similar study by M. Maiwulanjiang et al. showed that Song Bu Li decoction, a herbal remedy prepared by boiling with Nardostachyos Radix et Rhizoma, protected against cellular toxicity induced by tert-butyl hydroperoxide in cultured PC12 cells. They suggested that the protective effect of the herbal drug was mediated by activating the transcriptional activity of antioxidant response element.

Previous studies by Y.-F. Xian et al. have identified an active ingredient (isorhynchophylline) from Uncariae Ramulus cum Uncis by bioassay-guided fractionation which is effective in inhibiting neurotoxicity induced by beta-amyloid. In this special issue, the authors suggested that the protective effect of isorhynchophylline against beta-amyloid-induced cytotoxicity in PC12 cells was associated with the enhancement of p-CREB expression via PI3K/Akt/GSK-3*β* signaling pathway.

Apart from bioassay-guided isolation, high-throughput screening is another commonly used method to search for novel drugs. In recent years, a new method of drug discovery (*in silico* virtual screening) has been adopted by many researchers. By modelling the laboratory processes such as the binding of molecules to a receptor via computer algorithms, potential drugs can be identified and then confirmed by biological testing. This approach will greatly reduce laboratory works in high-throughput screening. In this special issue, Y. Wang et al. have identified twelve phytochemicals as acetylcholinesterase inhibitors by using *in silico* screening method. Subsequent biological tests conducted by the research team showed that acetylshikonin was the most effective compound among these acetylcholinesterase inhibitors in preventing apoptosis induced by hydrogen peroxide in neuronal SH-SY5Y and PC12 cells.

Parkinson's disease is the second most common neurodegenerative disorder which is characterized by the loss of dopaminergic neurons in the substantia nigra of ventral midbrain area. Tremor, rigidity, bradykinesia, and postural instability are the typical symptoms observed in patients suffering from the disease. 6-Hydroxydopamine is a neurotoxin which can selectively destroy dopamine-generating neurons in the brain. Therefore, neurotoxicity induced by 6-hydroxydopamine is commonly used as a model of Parkinson's disease. By using this model, X.-B. Meng et al. demonstrated that notoginsenoside R2, a triterpenoid isolated from Notoginseng Radix et Rhizoma (root and rhizome of *Panax notoginseng*), protected against neurotoxicity through the enhancement of phase II detoxifying enzymes which were triggered by the activation of MEK1/2-ERK1/2 pathways. Besides, a literature review by S.-V. More et al. indicated that a lot of chemical ingredients isolated from herbal medicine could be the potential drugs for treating Parkinson's disease.

The prevention of illness by maintaining homeostasis and enhancing body's defense is a major goal of herbal medicine. K.-Y. Zhu et al. showed that Kai-Xin-San, a herbal formula prescribed traditionally to treat stress-related psychiatric diseases, was able to stimulate the expression and secretion of neurotrophic factors in cultured astrocytes. These neurotrophic factors are playing important roles in maintaining the survival, growth, and differentiation of neurons. The depletion of neurotrophic factors can lead to neuronal death which contributes to the pathogenesis of neurodegenerative disorders. By using similar techniques, S.-L. Xu et al. demonstrated that a lot of flavonoids isolated from herbal materials were able to induce the synthesis and secretion of neurotrophic factors, including nerve growth factor, glial-derived neurotrophic factor, and brain-derived neurotrophic factor.

In the last special issue, we have mentioned that clinical trial is needed to provide supporting evidence for the application of herbal medicine on neurodegenerative disorders. In this issue, a clinical report by W. Pan et al. showed that Jiawei Sijunzi decoction, a six-herb herbal formula, delayed the development of amyotrophic lateral sclerosis in some patients. We still hope that large-scale, double-blind, placebo-controlled trial can be collected in the coming issues.

